# Supplementary material for: RADAR: A novel fast-screening method for reading difficulties with special focus on dyslexia
Source: PLoS One. 2017 Aug 11;12(8):e0182597. doi: 10.1371/journal.pone.0182597 (PMC5553666; doi:10.1371/journal.pone.0182597)
Supplement: S1 Appendix — The Bayesian analysis leading to the total score classifier. (PDF) [file pone.0182597.s001.pdf]

Appendix

The results obtained, were collected in a vector $\underline{N}$, and combined as follows to obtain the final reading score: First all the parameters in $\underline{N}$ were normalized to $\underline{Z}$ by subtracting the mean and dividing by the standard deviation. Then, the covariance matrices C_{contr}, C_{dysl} of the parameters $\underline{Z}$ were obtained for the control and the dyslexic populations respectively. Multivariate Gaussian distributions are generated for both the control and the dyslexic populations using these covariance matrices:

$$p(\underline{z}/control) = \frac{1}{\sqrt{2\pi \det(C_{contr})}} \exp(-\frac{1}{2}\underline{z}^T C_{contrl}^{-1}\underline{z}),$$

$$p(\underline{z}/dyslexic) = \frac{1}{\sqrt{2\pi \det(C_{dysl})}} \exp(-\frac{1}{2}\underline{z}^T C_{dysl}^{-1}\underline{z}),$$

These are taken to be the distributions of the vector of normalized parameters $\underline{Z}$ in each population.

To obtain the probability that an individual is in the control group versus the dyslexic group, we use the Bayes theorem to get

$$P(control/\underline{z}) = \frac{p(\underline{z}/control)p(control)}{p(\underline{z}/control)p(control) + p(\underline{z}/dyslexic)p(dyslexic)},$$

$$P(dyslexic/\underline{z}) = \frac{p(\underline{z}/dyslexic)p(dyslexic)}{p(\underline{z}/control)p(control) + p(\underline{z}/dyslexic)p(dyslexic)},$$

where the prior probabilities of an individual being in the control or dyslexic groups are given by the fraction of typical readers versus readers with dyslexia among all participants examined.

The score of an individual is the probability $P(control/\underline{N}) = P(control/\underline{z})$ of the individual. A score above 0.5 indicates that an individual has higher probability to be in the control group, while a score below 0.5 indicates that an individual has a higher probability to be in the dyslexic group (see Fig 4).
